# First report of unusual case of parasitism by *Amblyomma nodosum* (Neumann, 1889) in a yellow cururu toad (*Rhinella icterica*) in the Northeastern Brazilian Caatinga

**DOI:** 10.1590/S1984-29612024031

**Published:** 2024-06-28

**Authors:** Daniel Antônio Braga Lee, Darci Moraes Barros-Battesti, Paulo Vitor Cadina Arantes, Jovêncio Mateus Sada, Gustavo Seron Sanches, Marcos Rogério André, Victor Fernando Santana Lima

**Affiliations:** 1 Departamento de Patologia, Reprodução, e Saúde Única, Faculdade de Ciências Agrárias e Veterinárias – FCAV, Universidade Estadual Paulista – UNESP, Jaboticabal, SP, Brasil; 2 Laboratório de Doenças Parasitárias, Departamento de Medicina Veterinária do Sertão, Universidade Federal de Sergipe – UFS, Campus do Sertão, Nossa Senhora da Glória, SE, Brasil

**Keywords:** Tick, host, amphibian, Amblyomma nodosum, Carrapato, hospedeiro, anfíbio, Amblyomma nodosum

## Abstract

The *Amblyomma* genus (Arachnida: Ixodidae) is widely distributed in South America, with 34 species occurring in Brazil. *Amblyomma nodosum* Neumann 1889 is a species that predominantly feeds on Passeriformes during immature stages (larvae and nymphs) and anteaters (Myrmecophagidae) during adult stages. The aim of the present study is to report, for the first time, an unusual case of parasitism by adults of *A. nodosum* on a yellow cururu toad (*Rhinella icterica*) captured in the city of Nossa Senhora da Glória, Sergipe state (Northeastern Brazil) in the Caatinga biome, and also investigate the presence of DNA of *Rickettsia* in the collected material. DNA was extracted from all specimens collected (N=8) and subjected to PCR assays based on the tick 16S rRNA endogenous gene and *gltA* gene for *Rickettsia* sp. All samples (8/8; 100%) were positive for the 16S rRNA endogenous gene and two amplicons (obtained from one male and one female) were purified and sequenced. The BLASTn analysis of the sequences revealed a high degree of similarity (95-100%) with *A. nodosum* sequences previously deposited on GenBank, while the phylogenetic analysis clustered the sequences obtained in the same clade as *A. nodosum* sequences from Brazil.

## Resumo

O gênero *Amblyomma* (Arachnida: Ixodidae) é amplamente distribuído na América do Sul, com 34 espécies ocorrendo no Brasil. *Amblyomma nodosum* Neumann 1889 é uma espécie que se alimenta predominantemente de Passeriformes, durante os estágios imaturos (larvas e ninfas), e de tamanduás (Myrmecophagidae) durante os estágios adultos. O objetivo do presente estudo é relatar, pela primeira vez, um caso incomum de parasitismo por adultos de *A. nodosum* em um Sapo-cururu (*Rhinella icterica*), capturado na cidade de Nossa Senhora da Glória, estado de Sergipe (Nordeste do Brasil) na Caatinga, e também investigar a presença de DNA de *Rickettsia* no material coletado. DNA foi extraído de todos os espécimes (N=8) coletados e submetidos a ensaios de cPCR baseados no gene endógeno 16S rRNA de carrapatos e no gene *gltA* de *Rickettsia* sp. Todas as amostras (8/8; 100%) foram positivas para o gene endógeno 16S rRNA e dois amplicons (obtidos de um macho e uma fêmea) foram purificados e sequenciados. A análise BLASTn das sequências revelou um alto grau de similaridade (95-100%) com sequências de *A. nodosum* previamente depositadas no GenBank, enquanto a análise filogenética agrupou as sequências obtidas no mesmo clado das sequências de *A. nodosum* do Brasil.

## Introduction

Approximately 905 species belong to the order Ixodida, which includes ticks that specialize in blood feeding and can parasitize both cold-blooded and warm-blooded animals, including humans. To date, three families are part of the Ixodida order: Argasidae, Ixodidae and Nutalliellidae. The Ixodidae family, which is formed by the hard ticks, has the highest richness of species (~785 species) (Beati & Klompen, 2019; Guglielmone et al., 2023). Beyond the harm caused by infestations on domestic, wild and livestock animals, ixodids bear significant implications for public health since they may transmit many pathogens, including zoonotic agents, such as *Anaplasma phagocytophilum, Borrellia burgdorferi, Ehrlichia chaffeensis* and *Rickettsia rickettsii* (Boulanger et al., 2019; Rochlin & Toledo, 2020).

Among ixodid ticks, the *Amblyomma* genus stands out with a wide range of species (~136), of which 68 occur in the Neotropical Region (Guglielmone et al., 2021; Soares et al., 2023). In Brazil, a total of 34 *Amblyomma* species have been reported, including two recently described species (*Amblyomma romarioi* and *Amblyomma monteiroae*) (Martins et al., 2019; Dantas-Torres et al., 2019; Fuverki et al., 2021; Soares et al., 2023; Labruna et al., 2023).

The Neotropical species *Amblyomma nodosum* is distributed from Mexico to Argentina. In Brazil, this species has been reported in the North (states of Acre, Amazonas Pará, Rondônia, Tocantins), Northeast (states of Bahia, Ceará, Maranhão, Paraíba, Pernambuco), Central-West (states of Goiás, Mato Grosso do Sul, Mato Grosso), Southeast (states of Espírito Santo, Minas Gerais, Rio de Janeiro, São Paulo), and South regions (states of Paraná, Rio Grande do Sul, Santa Catarina) (Dantas-Torres et al., 2022; Labruna et al., 2023). It has been commonly found parasitizing wild mammals and birds in intact and well-preserved areas of Central and South America (Argentina, Belize, Bolivia, Colombia, Costa Rica, Guatemala, Honduras, Mexico, Nicaragua, Panama, Paraguay, Trinidad and Tobago, Venezuela) (Labruna et al., 2023; Guglielmone et al., 2021; Ogrzewalska et al., 2009). The main hosts of the adult ticks are anteaters belonging to the Myrmecophagidae family, although they can infest other hosts, such as armadillos (Chlamyphoridae) and three-fingered sloths (Bradypodidae) (Bechara et al., 2002; Guglielmone et al., 2021). Immature stages (larvae and nymphs) predominantly feed on Passeriformes, with a much larger variety of hosts for nymphs, compared with larvae (Guglielmone et al., 2021). The aim of the present study was to report for the first time the unusual parasitism of *A. nodosum* in a cold-blooded animal, a yellow cururu toad [*Rhinella icterica* Spix, 1824 (Anura: Bufonidae)] rescued in the Sergipe state, Northeastern Brazil (Caatinga biome). Because of a Spotted Fever Group *Rickettsia* was previously detected in adult ticks of *A. nodosum*, we also investigated the presence of DNA of this bacterium in the collected material. It is worth remembering that this tick species is associated with *Rickettsia parkeri* strain NOD (Ogrzewalska et al., 2009), although this strain has never been recorded causing spotted fever in humans (Moerbeck et al., 2018).

## Material and Methods

### Tick collection

Ticks were collected from one specimen of yellow cururu toad (*Rhinella icterica* Spix, 1824) sent for care at an outpatient clinic of the Federal University of Sergipe Veterinary Clinic School in 2023. The animal had been found in a residence located in urban land expansion area of the city of Nossa Senhora da Glória (-10° 13’ 5.99” S and -37° 25’ 13.01” W), in state of Sergipe, Northeastern Brazil, Caatinga biome. After clinical examination and visual inspection, manual collection of ectoparasites attached to the skin was carried out. All specimens were placed in 1.5 mL collection tubes containing absolute alcohol.

### Morphological identification and DNA extraction

The morphologic identification of the ticks obtained was performed using published morphological keys for ixodid ticks (Barros-Battesti et al., 2006). After the morphological identification, DNA was extracted from each tick specimen using the TRIzol™ (Invitrogen®, Thermo Scientific), following the manufacturer instructions. The DNA concentration and purity (260/280 ratio) was assessed using a spectrophotometer (Nanodrop®, Thermo Scientific). DNA was stored in DNAse/RNAse-free microtubes under -20 °C until PCR was done.

### PCR assays for endogenous tick 16S rRNA gene and *Rickettsia* sp. *gltA* gene

Initially, all samples were subjected to a PCR assay based on the tick endogenous 16S rRNA gene (Black & Piesman, 1994), in order to obtain sequences to molecularly confirm the morphological identification of the adult ticks. For this reaction, we used an *Amblyomma dubitatum* DNA sample as a positive control. Positive samples for the 16S rRNA assay were then subjected to a PCR based on the *gltA* gene for *Rickettsia* spp. (Labruna et al., 2004). For this reaction we used a *Rickettsia parkeri* DNA as a positive control. We used ultrapure water as a negative control for both assays.

All PCR products were subjected to horizontal electrophoresis on a 1% agarose gel stained with ethidium bromide (0,5 μL/mL) in TEB running buffer (pH 8.0; 44,58 M Tris-base, 0,44 M boric acid and 12.49 mM EDTA). A 100 base pair molecular weight marker (Life Technologies® 134) was used to determine the size of the amplified products. The results were visualized and analyzed using an ultraviolet light transilluminator, coupled to computer data analyses program (ChemiDoc MP Imaging System, BIO RAD® 136). Amplicons from the PCR based on the tick 16S rRNA gene were selected for sequencing and further BLASTn and phylogenetic analyzes.

### Purification and sequencing

The products amplified in the PCR assays were purified using the The Wizard® Genomic DNA Purification Kit (Promega), following the manufacturer instructions. The sequencing of amplified products was performed through an automatized technique based on the dideoxynucleotide chain termination method. The same oligonucleotide primers used in the PCR assays were used. The sequencing was performed in the ABI PRISM 3700 DNA Analyzer (Applied Biosystems) sequencer, at the Center for Biological Resources and Genomic Biology (CREBIO) located in the department of Technology at the Sao Paulo State University (UNESP-FCAV).

### BLASTn and phylogenetic analyses

The consensus sequences obtained were submitted to BLAST software (NCBI, 2024a) to check the percentage of similarity between the detected (sequenced) 16S rRNA sequences and those previously deposited in GenBank (NCBI, 2024b).

All consensus sequences saved in “FASTA” files were aligned with *Amblyomma* spp. sequences retrieved from GenBank, using the software MAFFT Version 7.0 (RIMD, 2024). The alignment saved in “FASTA” extension was converted into a “NEXUS” file using the Alignment Transformation Environment (ALTER) software (UVigo, 2024). The best evolutive model was chosen using the MrModelTest2 2.4 (Nylander, 2004) through the PAUP4* Version 4c software (Swofford, 2024).

A maximum likelihood analysis was performed using the IQ-TREE 2 software (IQ-TREE, 2024), using 10^3^ Ultrafast Bootstrap Replicates for the alignment. The resulting phylogenetic tree was rooted (via outgroups) and edited using both FigTree 1.4.4 software (Rambaut, 2024) and MEGA Version 11 (MEGA, 2024) softwares.

## Results

**Figure 1:**
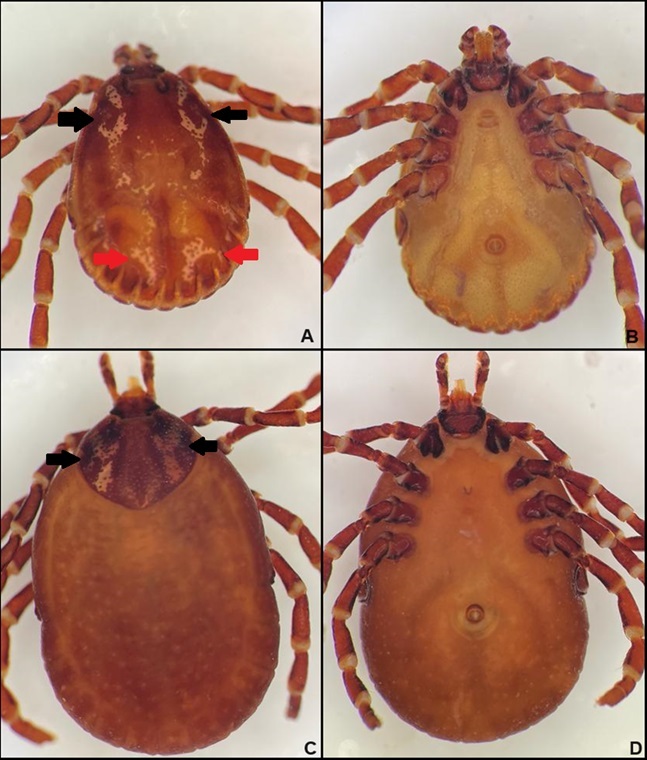
Morphological characteristics of *Amblyomma nodosum* ticks collected on a yellow cururu toad (*Rhinella icterica*) from Brazilian Caatinga. (A) Male, dorsal view. The black arrows indicate the J-shaped spots and the red arrows indicate the inverted U-shaped spots on the scutum (B) Male, ventral view; (C) Female, dorsal view. The arrows indicate the Y-shaped spots on the scutum; (D) Female, ventral view.

A total of eight adult ticks were collected from the toad, being one female (12.5%) and seven males (87.5%). Based on the morphological characteristics, all specimens were identified as *Amblyomma nodosum* (Figure 1).

**Figure 2:**
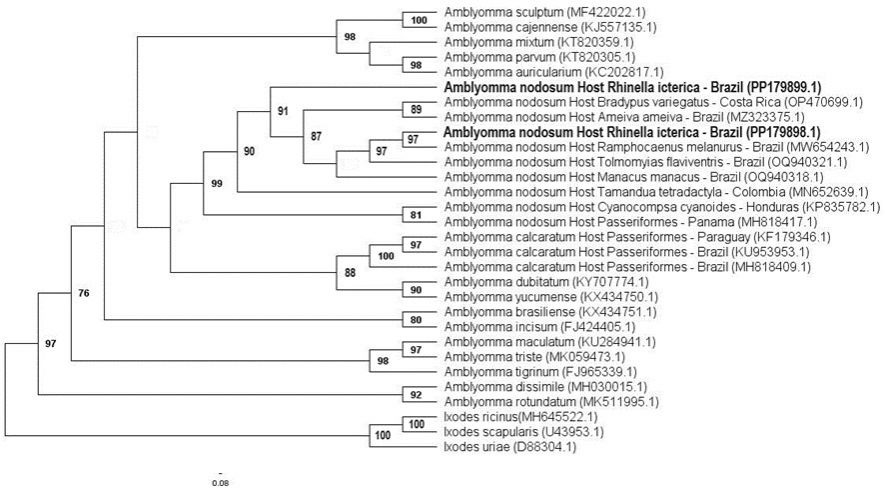
Phylogenetic tree based on an alignment of 428 bp length of *Amblyomma* spp. 16S rRNA sequences, using the Maximum Likelihood (ML) method and GTR+I+G as the evolutionary model. Sequences obtained in the present study are highlighted in bold. *Ixodes ricinus*, *Ixodes scapularis* and *Ixodes uriae* were used as outgroups.

All samples were positive in the PCR assay based on the endogenous 16S rRNA gene. Amplicons were selected from one female and one male with the strongest band intensities in the electrophoresis gel for sequencing. The BLASTn analyses of the two sequences (418 and 452-bp) confirmed the morphological identification by showing a high identity (95-100%) to *A. nodosum* sequences previously deposited on GenBank. The phylogenetic analyses clustered the obtained sequences in the same sub-clade as *A. nodosum* sequences detected in Xenarthra, *Bradypus variegatus* Schinz, 1825 and *Tamandua tetradactyla* (Linnaeus, 1758) from Costa Rica and Colombia, Passeriformes [*Manacus manacus* (Linnaeus, 1766), *Tolmomyias flaviventris* (Wied, 1831) and *Ramphocaenus melanurus* (Zimmer, 1937) and Squamata [*Ameiva ameiva* (Linnaeus, 1758)] from Brazil (Figure 2). We deposited our sequences under the accession numbers PP179898 and PP179899.

All samples were negative to the PCR for *Rickettsia* spp. based on the *gltA* gene.

## Discussion

This is the first study in the literature in which adult stages of *Amblyomma nodosum* ticks were collected from an amphibian, a yellow cururu toad (*Rhinella icterica*). In Brazil, the primary hosts for *A. nodosum* adults are the Collared Anteater (*Tamandua tetradactyla*) and the Giant Anteater (*Myrmecophaga tridactyla*), both of which can be found in all Brazilian biomes (Szabó et al., 2019). Although there is a lack of reports of *A. nodosum* ticks parasitizing hosts other than anteaters, in a few cases those ticks have been reported parasitizing unconventional hosts. Unexpected parasitism by *A. nodosum* has been reported in mammals, including a capybara [*Hydrochoerus hydrocharesis* (Rodentia: Caviidae)] in the state of Rondônia, northern Brazil (Amazon Rainforest biome) and a domestic dog [*Canis familiaris* (Carnivora: Canidae)] from the state of Espírito Santo, southeastern Brazil (Atlantic Forest biome) (Fuverki et al., 2021; Mazioli et al., 2012). On the other hand, *A. nodosum* immature specimens are commonly found on birds, especially Passeriformes (Guglielmone et al., 2021). Additionally, *A. nodosum* adult and immature ticks have been reported parasitizing reptiles, including males and females parasitizing a boa constrictor [*Boa constrictor* Linnaeus, 1758 (Squamata: Boidae)] in the Central-West region of Brazil (Mendoza-Roldan et al., 2020), and a female and immature stages (larvae and a single nymph) parasitizing a green ameiva [*Ameiva ameiva* (Squamata: Teiidae)] in the Central Amazonic region (Dantas-Torres et al., 2022).

Previous studies have shown that, under laboratory conditions, *A. nodosum* exhibit a high degree of dependence on their hosts and environment for development (Pinheiro et al., 2015). Although the records of unexpected parasitism by this tick species is considered incidental, there could be a potential relation between the selection of a host by this tick species and various ecological features, such as habitat specificity, host availability, and type of cycle of the ticks and environmental conditions (e.g. temperature, humidity) (Nava & Guglielmone, 2013). Considering that while collared anteaters and giant anteaters are found across all Brazilian biomes (including Caatinga), the unusual parasitism reported herein might be associated to primary host scarcity. The yellow cururu toad was captured in a land expanding area of the Nossa Senhora da Glória city (between urban and wild areas), where many intact habitats were dismantled by urban development, possibly causing a displacement of the main hosts and potentially causing the ticks to feed on unconventional hosts.

The ticks collected in the present study were identified as *A. nodosum* based on morphological and molecular methods. It is important to highlight that *A. nodosum* life cycle stages (especially larvae and nymphs) can be misidentified as *A. calcaratum*, and vice versa, due to morphological similarities (Guglielmone et al., 2021). Our phylogenetic analysis demonstrated that the sequences we obtained shared a clade with *A. calcaratum* sequences from Paraguay, Brazil and Panama, but were positioned in the same sub-clade of *A. nodosum* sequences reported in Passeriformes, Xenarthra and Squamata from Brazil.

Although we did not detect the presence of *Rickettsia* sp. in the analyzed ticks, rickettsial infections have already been reported in *A. nodosum* ticks collected from different hosts in Brazil. *Rickettsia parkeri* strain NOD, a Spotted Fever Group *Rickettsia*, has been detected in adult ticks (reared from nymphs) collected from wild passeriform birds in the Atlantic Forest biome (Ogrzewalska et al., 2009) and from adults collected on *M. tridactyla* and *T. tetradactyla* from Southeastern and Central-Western Brazil (Atlantic Forest and Cerrado Biomes) (Szabó et al., 2019). Although there are no reports of *A. nodosum* ticks feeding on humans, a wide variety of Passeriformes hosts (~83 spp.) can act in the maintenance and spread of rickettsial infections to other hosts (vertebrates and other tick species and blood-feeding arthropods), thus participating in the enzootic cycle of rickettsial diseases (Almeida et al., 2013; Guglielmone et al., 2021). Considering the wide distribution of their main hosts (Myrmecophagidae and Passeriformes), and reports of unexpected hosts in different biomes, future studies should be performed aiming at investigating microorganisms associated to *A. nodosum* ticks to fully understand their role in the epidemiology of vector-borne infections.

## Conclusion

The present study reported, for the first time, the unusual parasitism by *A. nodosum* males and females on an amphibian, a yellow cururu toad (*Rhinella icterica*), captured in the state of Sergipe within the Caatinga biome. In addition to recording the occurrence of parasitism in a previously unreported host, we also provided phylogenetic information for the 16S rRNA gene fragment. Further studies aiming at understanding the ecological drives for the interaction between *A. nodosum* species and their hosts are much needed, in order to deeply understand tick-host dynamics and their association with pathogenic agents.
